# Mapping Trends and Hotspots Regarding the Use of Ultrasound in Emergency Medicine: A Bibliometric Analysis of Global Research

**DOI:** 10.3389/fpubh.2021.764642

**Published:** 2021-12-24

**Authors:** Sheng Wang, Demeng Xia, Zhentao Zhang, Jingli Zhang, Wenhao Meng, Yanping Zhang, Shuogui Xu

**Affiliations:** ^1^Department of Emergency, Changhai Hospital, Naval Medical University, Shanghai, China; ^2^Health Clinic, People's Liberation Army Unit 91666, Zhoushan, China; ^3^Department of Clinical Medicine, Naval Medical University, Shanghai, China; ^4^Department of Neurology Rehabilitation, Qingdao Women and Children's Hospital, Qingdao University, Qingdao, China; ^5^Department of Clinical Medicine, Wei Fang Medical University, Wei Fang, China; ^6^Nanjing Comprehensive Stroke Center, Nanjing Brain Hospital Affiliated to Nanjing Medical University, Nanjing, China

**Keywords:** ultrasound, emergency medicine, pulmonary embolism, point-of-care ultrasound, bibliometrics

## Abstract

**Objective:** Diagnostic tools in emergency medicine have been widely studied. As a non-invasive and quick tool, ultrasound plays a role in the field of emergency medicine. Thus, it is significant to understand the global scientific output of this topic. An analysis of publications on the use of ultrasound in emergency medicine over the past decade was performed and summarized to track the current hotspots and highlight future directions.

**Methods:** Globally relevant publications on ultrasound in emergency medicine from 2009 to 2020 were extracted from the Web of Science collection database. VOSviewer software and CiteSpace were employed to visualize and predict the trends in the research on the topic.

**Results:** The overall volume of global publications is on the rise**;** furthermore, the United States published the most publications in this field and had the most citations and H-index. *University of California at San Francisco* in the United States has most publications in terms of institutions. *The American Journal of Emergency Medicine* published the most papers related to ultrasound in emergency medicine in terms of journals. Pulmonary embolism was once the main research direction, and importantly, “point-of-care ultrasound” was determined to be a new research hotspot.

**Conclusion:** Altogether, the number of publications on ultrasound in emergency medicine will rise in the future. In addition, the findings reported here shed new light on the major progress on ultrasound in emergency medicine, which may be mutually cooperative in various fields. Moreover, this bibliometric study provides further indications for the topic of “point-of-care ultrasound”.

## Introduction

With the advancement of medical technology, ultrasound, as a non-invasive and convenient diagnostic tool, has been widely applied in the field of medical treatment, such as for the diagnosis of pleural effusion (1), tumor metastasis (2), and thrombosis (3). More importantly, unlike CT and MRI, ultrasound can be performed at the bedside, avoiding secondary injuries incurred while moving patients. The convenience of ultrasound makes it possible to use it to triage patients with suspected cases of coronavirus 2019 (COVID-19) (4). As a developing clinical discipline, emergency medicine includes a breadth of care from bystander resuscitation to emergency medical services transfer, treatment in intensive care units and many other aspects (5). With regard to the diagnosis of emergency diseases such as acute kidney stones (6), the rupture and hemorrhage of parenchymal organs (7, 8) and respiratory failure (9), ultrasound still plays an indispensable role. Moreover, it is not convenient to move critically ill patients in the intensive care unit, and bedside ultrasound is helpful to assess disease progression. Therefore, researchers and institutions continue to expand the application of ultrasound in the field of emergency medicine, but the details of current research trends have not yet been reported or tracked.

Bibliometrics is a type of qualitative and quantitative scientific evaluation. Through a comprehensive analysis of the author, country, H index, citation amount, publication time and other issues of the selected relevant articles, the contributions of academic groups and individual researchers were evaluated objectively. Similarly, through the integration and analysis of the keywords of the included articles, the keywords with high frequency and the keywords newly emerged in recent years, which were treated as hotspots, were selected to provide supporting evidence with regard to future development trends (10). Based on the studies obtained from the Web of Science (WOS), this study used bibliometrics to comprehensively analyse the progress of research on the use of ultrasound in the field of emergency medicine. Moreover, future research directions and hotspots were also predicted.

## Materials and Methods

### Data Sources and Search Strategies

**Figure 1:**
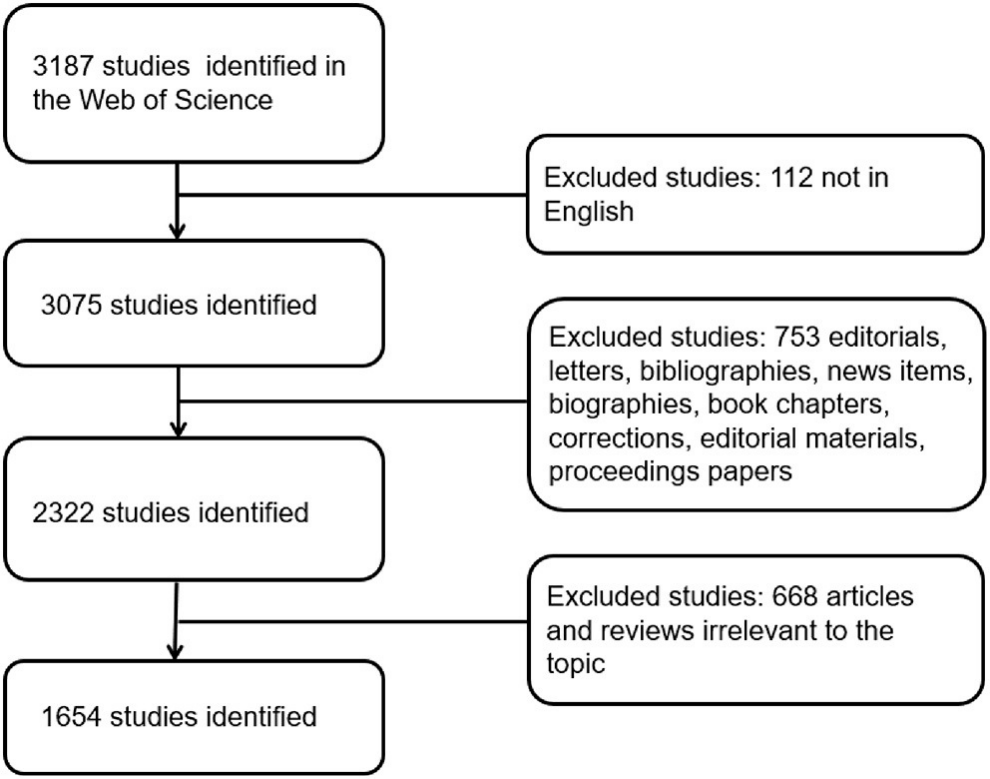
Flow diagram of the inclusion process. The detailed process of screening and enrolment (irrelevant articles were manually screened by two authors through abstracts and full texts, and articles irrelevant to the topic were excluded).

The online database Science Citation Index-Expanded (SCI-E) of Thomson Reuters' WOS is one of the most suitable tools for collecting and processing data; therefore, we obtained all studies published from 2009 to 2020 from this database. To avoid the daily update of the database to bring about changes in the included articles, we completed the search on October 6, 2020. We followed these steps to retrieve all relevant studies: TS = [(ultrasound) OR ultrasonography) OR (ultrasonic) OR (sonography)] AND Web of Science Categories = Emergency AND Language = English. Our study only included original articles, and other types of studies were excluded. Finally, articles irrelevant to the topic were filtered manually. The process was carried out by two authors, and if there was disagreement over the inclusion of a paper, the final decision was made by the experienced corresponding author. The specific processes of enrolment and selection are shown in Figure 1.

### Data Collection and Processing

All data (titles, keywords, authors, countries and regions of origin, institutions, published journals, publication dates, H-index, sum of citations, and so on) were extracted from the publications by two authors (WS and XDM). Microsoft Excel 2016 (Redmond, Washington, USA), GraphPad Prism 8 (GraphPad Prism Software Inc., San Diego, CA), VOSviewer version 1.6.12 (Leiden University, Leiden, the Netherlands), CiteSpace version 5.6. R5 64 bit (Drexel University, Philadelphia, PA, USA), and the Online Analysis Platform of Literature Metrology (http://bibliometric.com/) were used for presenting, analyzing and describing the data. Meanwhile, the World Bank website (https://www.worldbank.org/) was used to retrieve the gross domestic product (GDP).

### Bibliometric Analysis

A number of studies were collected from Thomson Reuters' Web of Science (WOS), especially those focused on biomedicine. Therefore, all included publications were collected from the WOS. The H index means that a researcher or a country has published at least H papers, and each paper has been cited in other publications at least H times; this is a useful metric for assessing scientific achievements (11). Relative research interest (RRI) was defined as the number of publications in a particular research field divided by the total number of publications across all fields per year. The impact factor (IF) was provided by Journal Citation Reports (JCRs) published in 2020. It has been widely accepted that the H-index, RRI, and IF all play important roles in evaluating the scientific research impacts of a researcher or a country, especially in recent years (12).

The science mapping software tool VOSviewer, which is a program developed for constructing and viewing bibliometric maps of authors, journals or keywords based on co-citation data, is applied to the analysis of countries, authors, and keywords in our research. While CiteSpace is a Java application for analyzing and visualizing co-citation networks, its primary goal is to facilitate the analysis of emerging trends in a knowledge domain, and it can be used for keyword clustering, which is helpful to summarize the general research direction (12).

## Results

**Figure 2:**
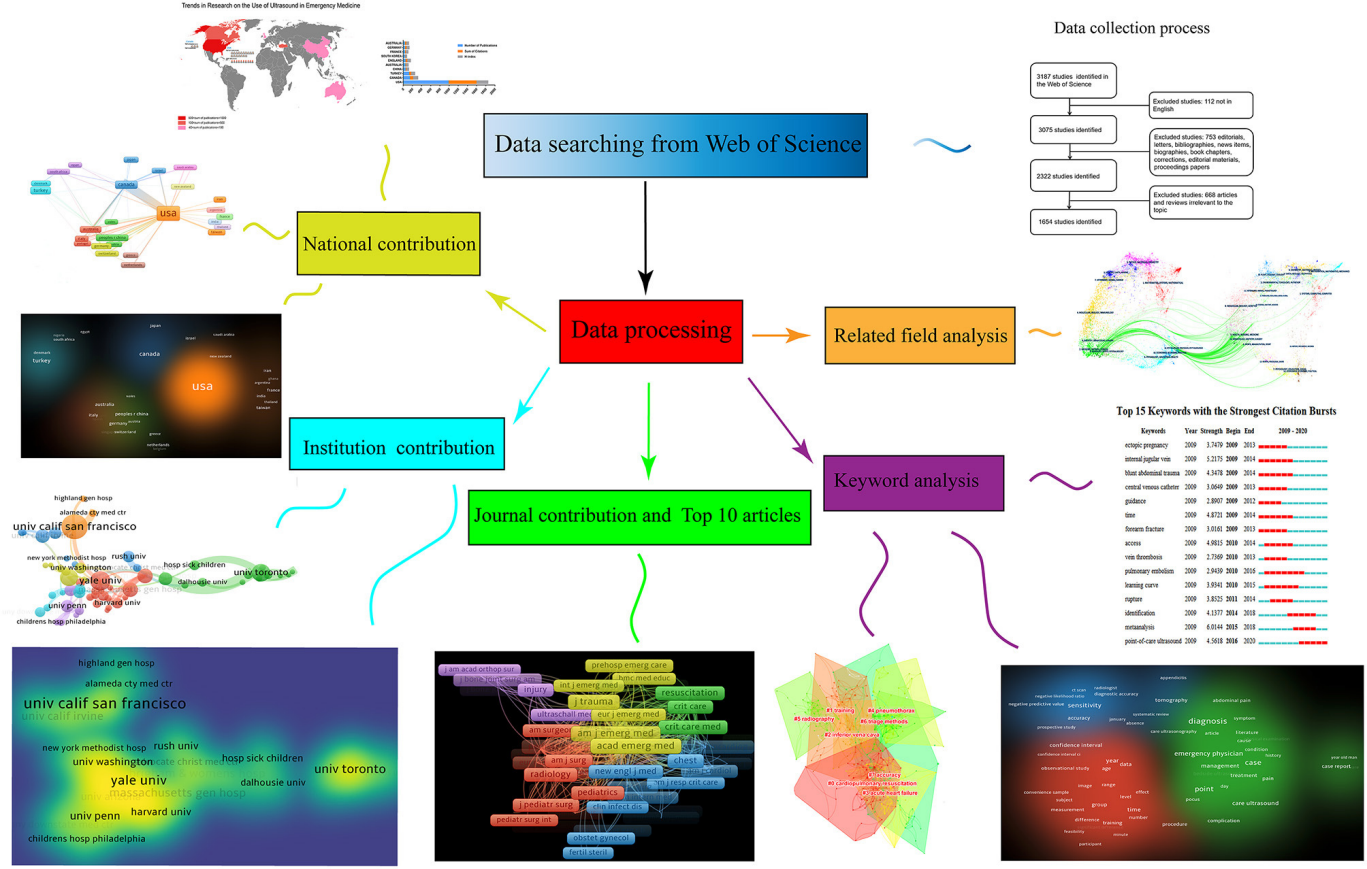
Flow diagram of five aspects of the use of ultrasound in emergency medicine. Contributions of countries, contributions of different journals and top 10 articles, contributions of different institutions, keywords and related fields were listed.

In all, 1,654 articles (112 non-English articles, 753 articles did not meet the type requirements, and 668 articles were not related to the topic were excluded) published from 2009 to 2020 met our inclusion criteria. By analyzing and summarizing the data, the five aspects of contributions of countries, contributions of different journals and top 10 articles, contributions of different institutions, keywords and related fields will be presented in the results. The specific processes are shown in Figure 2.

### Contributions of Countries to Global Publications

**Figure 3:**
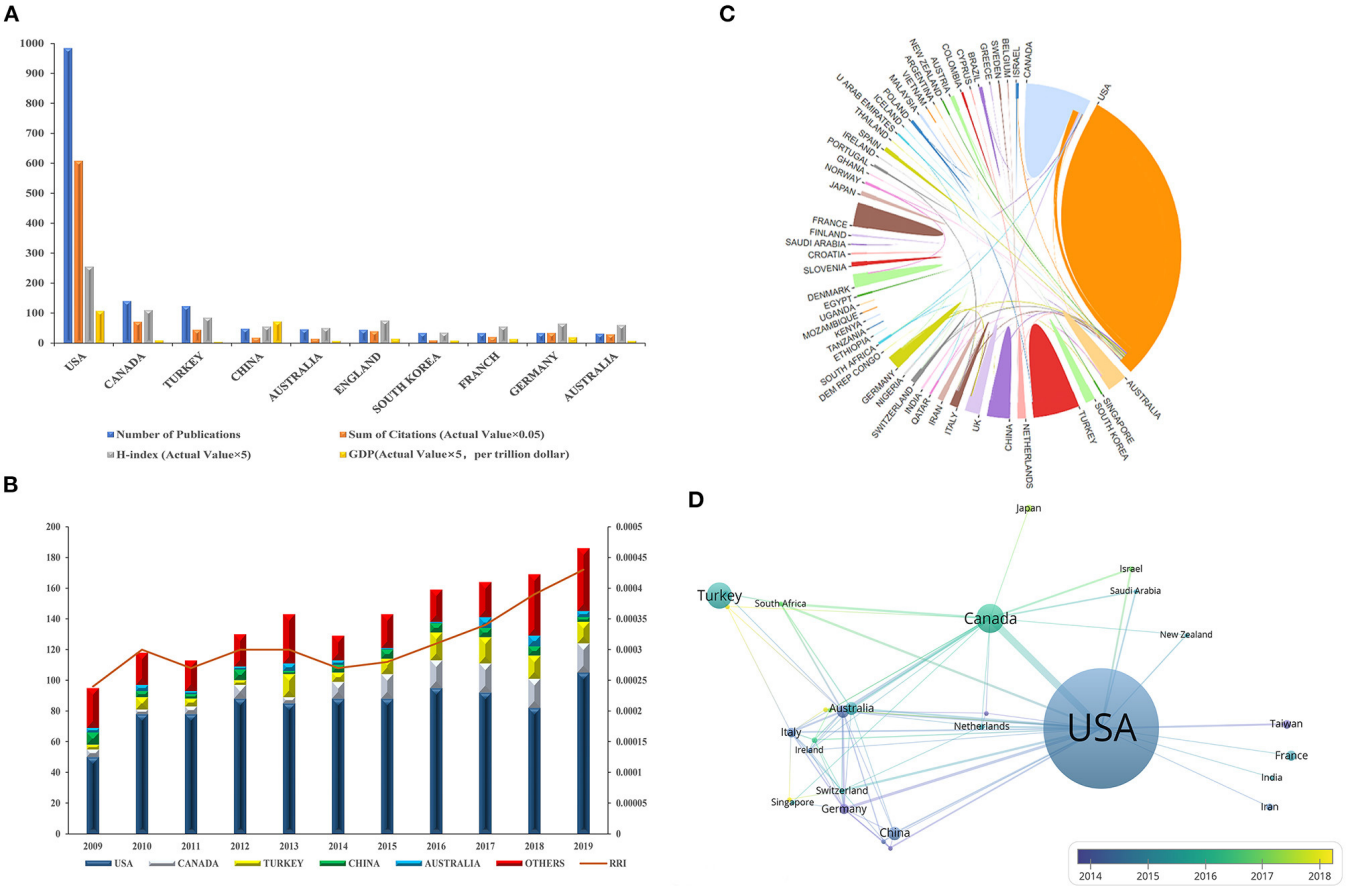
Contributions of different countries/regions to research on the use of ultrasound in emergency medicine and the cooperation network of countries/regions with regard to research on the use of ultrasound in emergency medicine. **(A)** The number of publications, citation frequency (×0.05), H-index (×5) and GDP (×5, per trillion dollar) in the top 10 countries or regions; **(B)** The number of publications worldwide and the time course of the RRI in the use of ultrasound in emergency medicine; **(C)** The cooperative relations between countries/regions were visualized; **(D)** The network of cooperative relations between countries/regions was established with VOSviewer.

We selected the country of the first corresponding author as the country to which this article belongs. The United States ranked first in the number of publications with 984 (27.9%), which accounted for more than half of the total publications, followed by Canada with 140 (8.5%) and Turkey with 123 (7.4%) (Figure 3A). According to the Journal Citation Report from the WOS database, all articles related to the use of ultrasound in emergency medicine had been cited 18,542 times since 2009 (15,545 times without self-citations), with an average citation frequency of 11.21 times per paper. The United States accounted for 65.6% of the total citations, i.e., 12,157 citations (10,782 times without self-citations), and had an H-index of 51. The numbers of citations from Canada and Australia were 1,416 (1,357 times without self-citations) and 880 (803 times without self-citations), with H-indices of 22 and 17, and these countries ranked second and third, respectively (Figure 3A).

By comparing the number of papers published per year, we found that most papers were published in 2019, when there were 186 publications (11.2%) (Figure 3B). When considering publications in all fields, the global interest in the use of ultrasound in emergency medicine measured by the RRI started to increase in 2015 and increased to nearly 0.045% in 2019, when it peaked (Figure 3B). We could predict that the growth trend in the number of publications in this field will accelerate in the future. The cooperative relations between countries were also visualized (Figures 3C,D). As the top two countries with the most publications, the United States and Canada cooperated the most in this field, and the United States was also connected with many other countries.

### Contributions of Different Institutions to Publications

**Figure 4:**
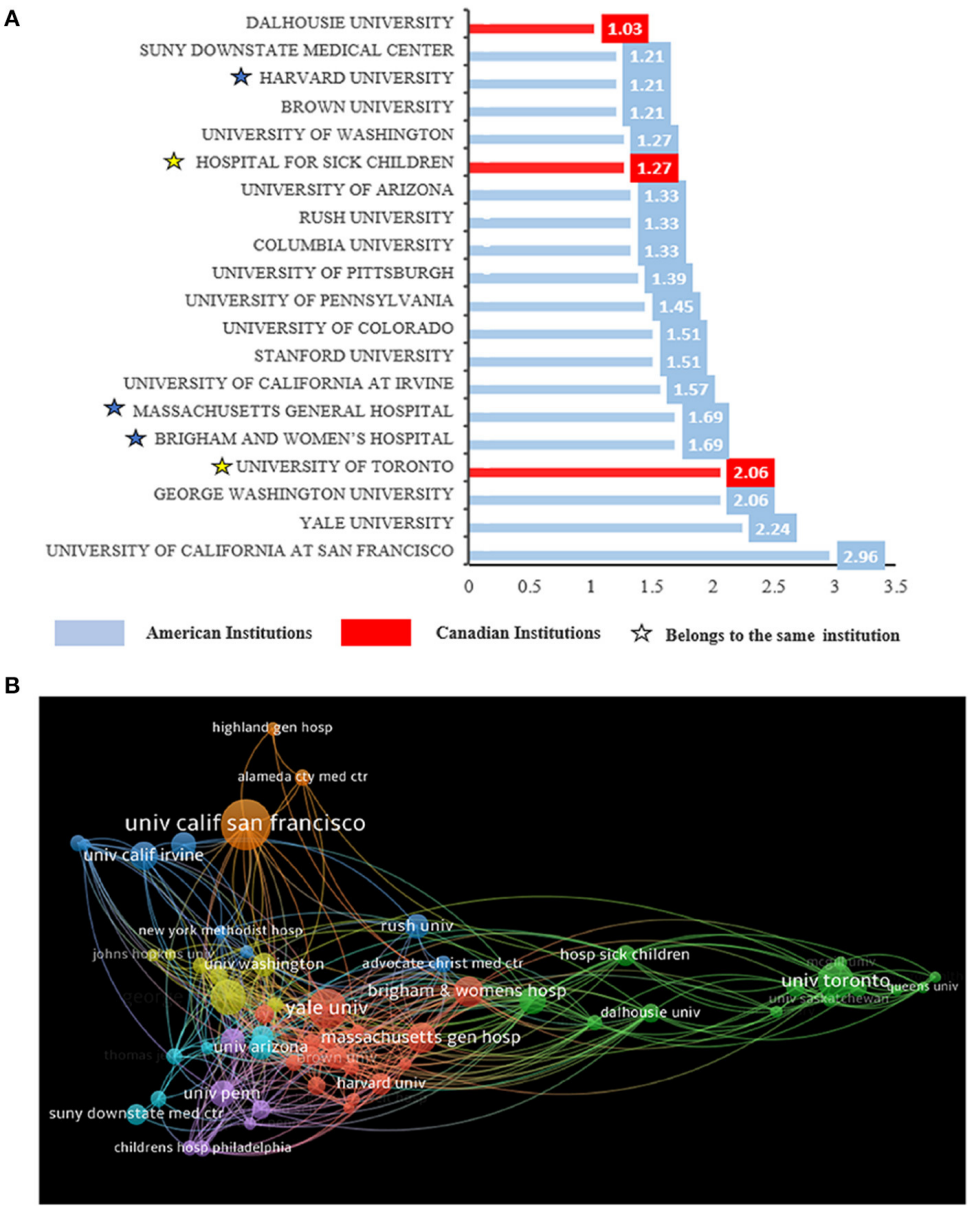
The distribution of institutions engaged in research on the use of ultrasound in emergency medicine. **(A)** Top 20 institutions by number of publications. Numbers represent the percentage of publications, with blue representing institutions in the United States and red representing institutions in Canada. Asterisks of the same color indicate that such organizations belong to a same organization. **(B)** The network of institutions produced in VOSviewer. The size of circles reveals the number of publications.

The University of California at San Francisco in the United States had the most publications among institutions worldwide in this field, with 55 papers, which accounted for 2.96% of all publications. Seventeen of the top 20 institutions in this field are in the USA; the other three institutions are in Canada, and Canada ranks second in terms of publications (Figure 4A).

In Figure 4B, the size of the ball represents the number of publications for the institution, and the top three institutions are the University of California at San Francisco, Yale University, George Washington University (Figure 4B). All three are from the United States. Asterisks of the same color indicate that such organizations belong to a same organization, after inspection, Brigham and Women's and Massachusetts General Hospital are both Harvard School affiliated hospitals, and Hospital for Sick Children is University of Toronto affiliated hospital. In fact, Harvard School in the United States had the most publications among institutions worldwide in this field, which accounted for 4.59% of all publications.

### Contributions of Different Journals to Publications and the Top 10 Articles

**Figure 5:**
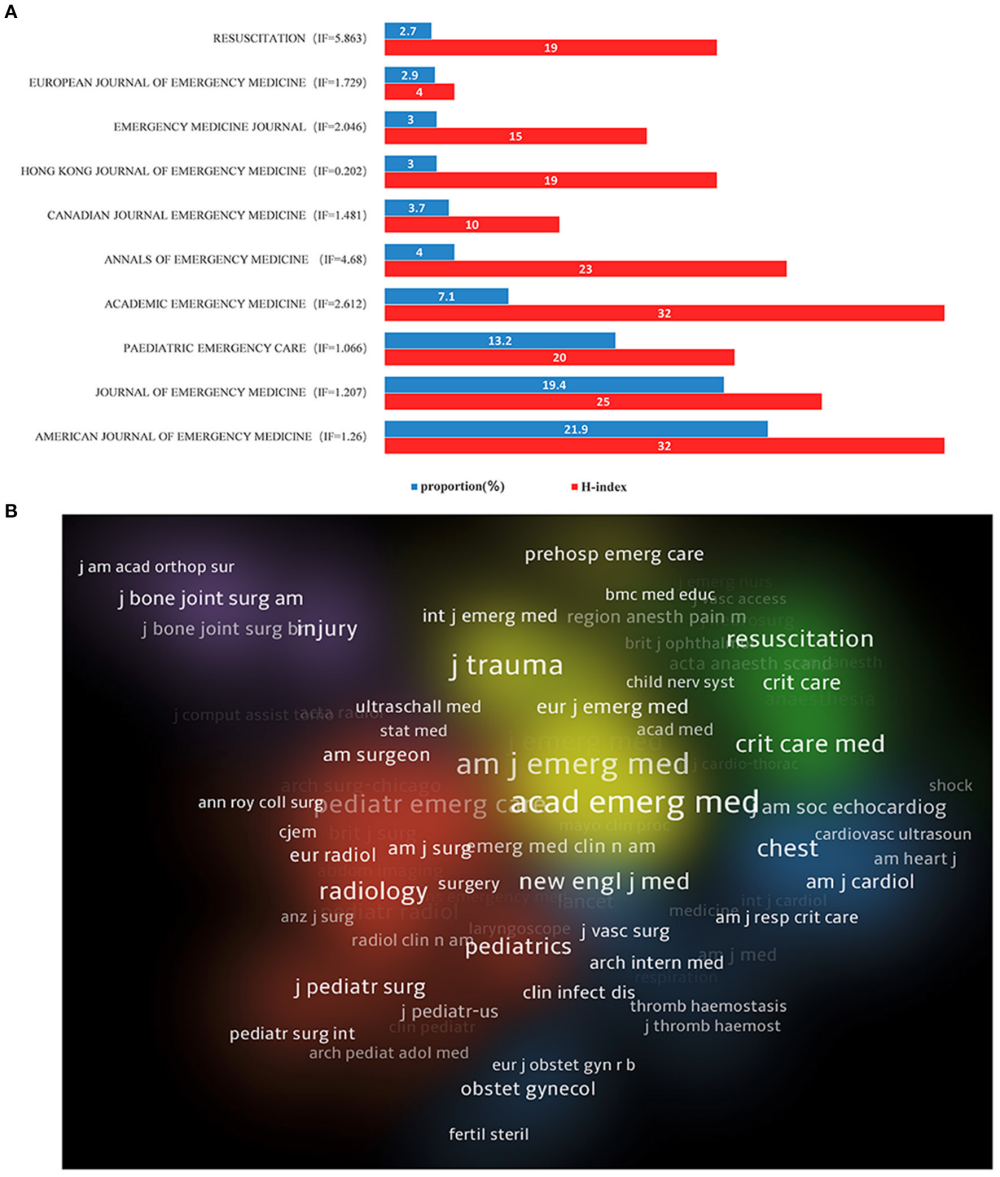
The distribution of journals engaged in research on the use of ultrasound in emergency medicine. **(A)** Top 10 journals by number of publications, blue bar representing the proportion and red bar representing H-index **(B)**. The network of institutions produced in VOSviewer, the size of circles reveals the H-index.

As shown in Figure 5A, more than half of the papers on this topic were published in the top three journals (903, 54.5%). Most papers were published in the *American Journal of Emergency Medicine* (IF = 1.290), with 363 records. The *Journal of Emergency Medicine* (IF = 1.207) ranked second in terms of the number of publications. The journals ranked first and second in terms of IF were *Resuscitation* (IF = 5.863) and *Annals of Emergency Medicine* (IF = 4.68), which had 45 and 66 publications on the use of ultrasound in emergency medicine and ranked tenth and fifth in the number of publications, respectively. Meanwhile, the H-index values of the *American Journal of Emergency Medicine* (32) and *Academic Emergency Medicine* (32) led them to be ranked first (Figure 5B). The top 10 journals with the most publications on the use of ultrasound in emergency medicine are listed in Figure 5A.

The most-cited article about the use of ultrasound in emergency medicine worldwide is *The RUSH Exam: Rapid Ultrasound in Shock in the Evaluation of the Critically Ill*, which was published by Perera, P in the United States, and this article was cited 227 times. Unsurprisingly, of the top 10 most-cited articles in this field, the American authors published half. It is worth mentioning that most of the top 10 most-cited articles were published in 2009 and 2010, and there were four articles published in Academic Emergency Medicine (Table 1).

**Table 1:** Top 10 most-cited papers related to the use of ultrasound in emergency medicine.

**Title**	**Corresponding authors**	**Journal**	**Publication year**	**Total citations**	**Corresponding author's country**
The RUSH Exam: Rapid Ultrasound in SHock in the Evaluation of the Critically Ill	Perera, P	Emergency Medicine Clinics of North America	2010	227	USA (13)
Focused echocardiographic evaluation in life support and pen-resuscitation of emergency patients: A prospective trial	Breitkreutz, R	Resuscitation	2010	221	Germany (14)
Emergency Department Bedside Ultrasonographic Measurement of the Caval Index for Non-invasive Determination of Low Central Venous Pressure	Merchant, RC	Annals of Emergency Medicine	2010	163	USA (15)
Emergency Thoracic Ultrasound in the Differentiation of the Etiology of Shortness of Breath (ETUDES): Sonographic B-lines and N-terminal Pro-brain-type Natriuretic Peptide in Diagnosing Congestive Heart Failure	Liteplo, AS	Academic Emergency Medicine	2009	153	USA (16)
Sensitivity of Bedside Ultrasound and Supine Anteroposterior Chest Radiographs for the Identification of Pneumothorax After Blunt Trauma	Stone, MB	Academic Emergency Medicine	2010	138	USA (17)
Point-of-care Ultrasonography for the Diagnosis of Acute Cardiogenic Pulmonary Edema in Patients Presenting With Acute Dyspnea: A Systematic Review and Meta-analysis	Cortellaro, F	Academic Emergency Medicine	2014	121	Italy (18)
Randomized Controlled Trial of Ultrasound-Guided Peripheral Intravenous Catheter Placement Vs. Traditional Techniques in Difficult-Access Pediatric Patients	Barbic, D	Pediatric Emergency Care	2009	114	Canada (19)
Lung ultrasound is an accurate diagnostic tool for the diagnosis of pneumonia in the emergency department	Doniger, SJ	Emergency Medicine Journal	2012	112	USA (20)
Tracheal rapid ultrasound exam (TRUE) for confirming endotracheal tube placement during emergency intubation	Lien, WC	Resuscitation	2011	103	China (21)
Resident Training in Emergency Ultrasound: Consensus Recommendations from the 2008 Council of Emergency Medicine Residency Directors Conference	Parlamento, S	Academic Emergency Medicine	2009	102	Italy (22)

### Analysis of Keywords in Publications on the Use of Ultrasound in Emergency Medicine

**Figure 6:**
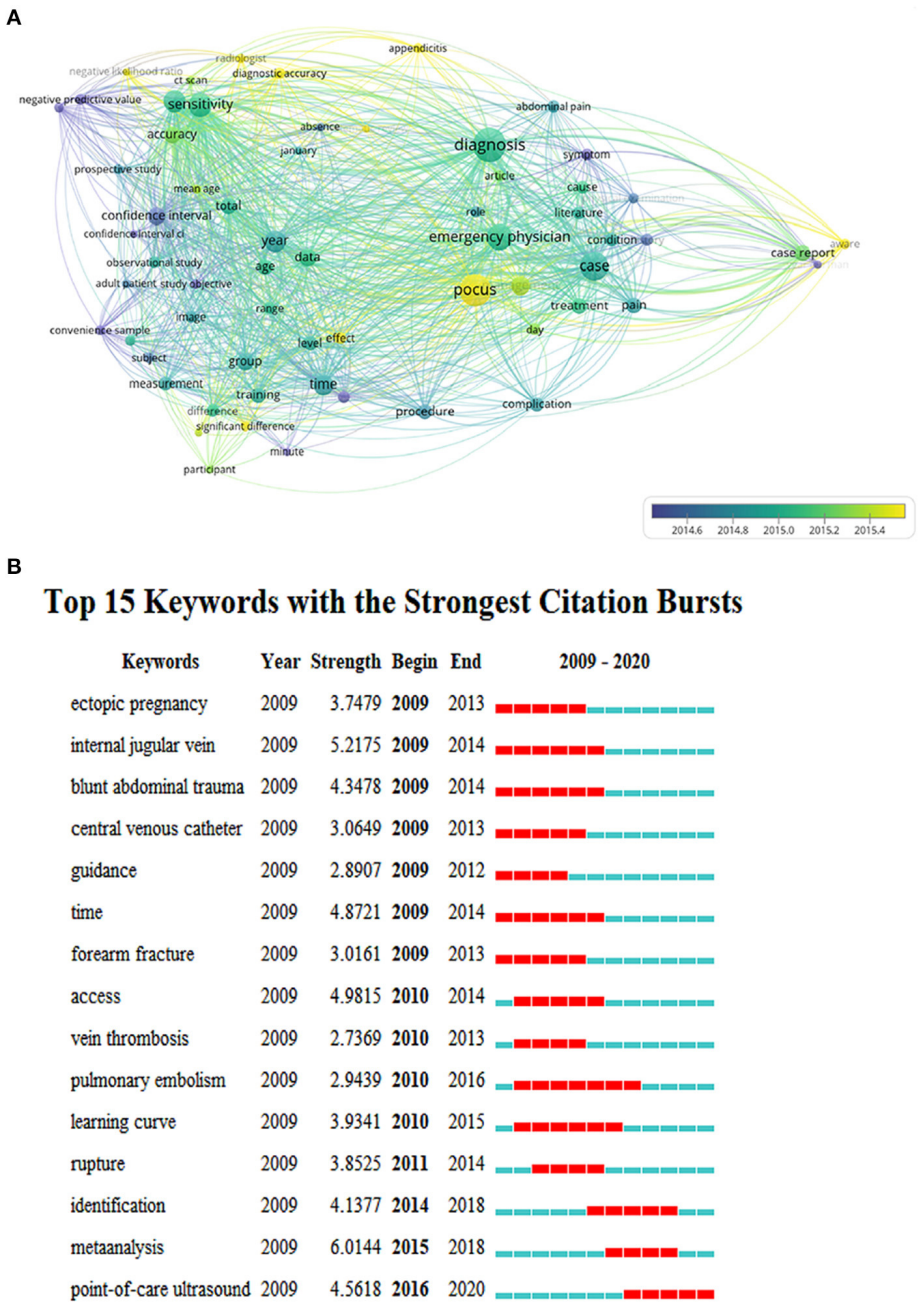
The cooccurrence analysis of all keywords in the publications on the use of ultrasound in emergency medicine. **(A)** Mapping of the keywords in the field of the use of ultrasound in emergency medicine. The size of the circle represents the frequency with which keywords appear. The distribution of keywords is presented according to the average time of appearance. The blue color represents an early appearance, and the yellow color represents a late appearance. **(B)** The top 15 keywords were cited the most frequently from 2009 to 2020 and have received continuous attention for a period of time. The red bars represent frequently cited keywords during this time period. The green bars represent infrequently cited keywords.

We analyzed the keywords extracted from 1,677 publications using VOSviewer. As shown in Figure 6A, 62 keywords, defined as terms that occurred more than 56 times within titles and abstracts in all papers during the analysis process, were frequently mentioned. The top three keywords are listed here: diagnosis (664 times), POCU(605 times), and case (514 times). Detailed data on the cooccurrence of all included keywords are presented in Figure 6A. Compared with the keywords that appear most frequently, recent keywords can represent current research hot spots, which will be more attractive to us. VOSviewer colored all keywords according to the average time the word appeared. Specifically, the blue color indicates that the word appeared relatively early, while the yellow color indicates a more recent appearance. After keyword processing, we found that “POCUS (point-of-care ultrasound)” was the latest among 62 keywords, and it appeared in April 2017.

From all 62 keywords, we compiled a list of the 15 keywords that last the longest time (Figure 6B). More recently, the newest salient keyword in our research was “POCUS (point-of-care ultrasound),” which was highlighted from 2016 to 2020 for ~5 years. “Pulmonary embolism” lasted the longest (7 years) from 2010 to 2016. This indicates that “pulmonary embolism” has been the focus of previous research, and since 2017, “POCUS” has gradually become the focus of research in this field.

### Related Field Analysis of the Use of Ultrasound in Emergency Medicine

**Figure 7:**
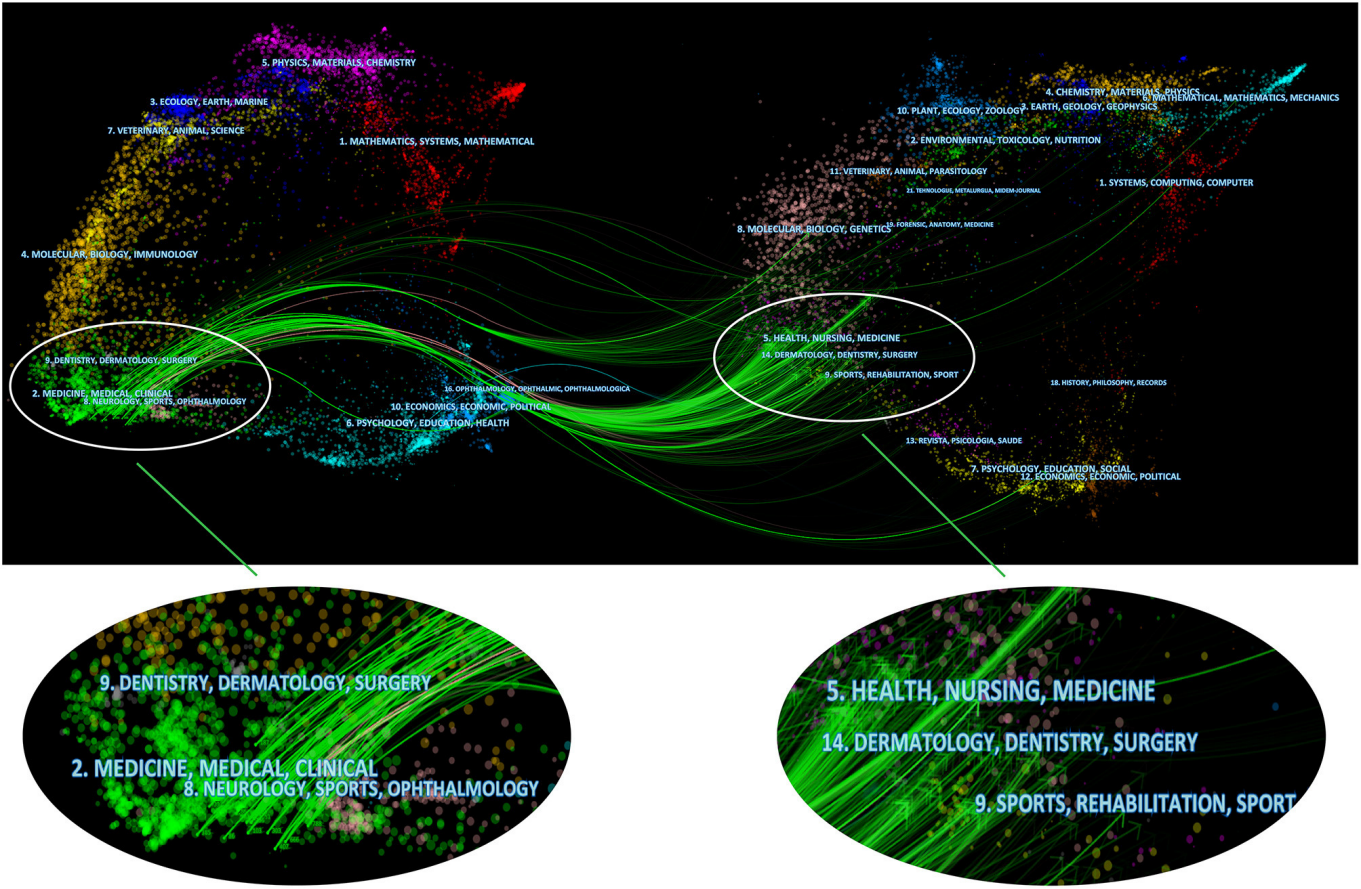
Related fields of the use of ultrasound in emergency medicine. The left side represents the fields of articles included in the study, and the right side represents the fields of references of articles.

In Figure 7, the 1,677 articles we included in our research can be mainly divided into three fields, one of which includes medicine, medical and clinical; the second field includes dentistry, dermatology and surgery; and the third field includes neurology, sports, ophthalmology. In addition, the references of these 1,677 articles were mainly distributed in the following three fields, one of which includes healthy, nursing and medicine, the second filed includes sports and rehabilitation and the third filed includes health, nursing and medicine. We found that ultrasound research in the emergency medicine field mainly involved subdisciplines in the field of clinical medicine, such as dentistry, dermatology surgery neurology and ophthalmology. The development of this field is related to the field of medicine, such as nursing, sports, and rehabilitation.

## Discussion

### Trends in Research on the Use of Ultrasound in Emergency Medicine

In terms of the publication volume of all countries, the United States contributed the most (Figure 3). Strong economic strength is supported by science and technology, so we speculated that the reason for this phenomenon is related to the index of the country's GDP; in this aspect, the United States ranked first (Figure 3A). At present, advanced ultrasound technology is often used in the United States and other developed countries; for example, musculoskeletal ultrasound imaging has promise for the diagnosis of emergency trauma, and the United States has already studied its use in the aspects of soft tissue infections, joint effusions, and long bone fractures (23). Similarly, complete systems of emergency medicine emerged in countries with stronger economies early, such as the guidelines for cardiopulmonary resuscitation (24), which were also contributed to by researchers from the United States. Whether in the field of emergency care or ultrasound, developed countries with advanced technology are better able to conduct research in this field. The country contact map based on WOS data shows that the United States has connections with many countries in the field, especially Canada (Figure 3C), and these two countries are the countries with the most publications, which also reminds researchers that mutual cooperation plays an important role in advancing the development of this field. In regard to research institutions, the results are also significantly influenced by country, 17 the top 20 institutions are from the United States, and the remaining three are from Canada (Figure 4A).

The published articles with the highest citation frequencies are associated with academic impacts in a certain field. Detailed information regarding the top 10 most frequently cited publications on the use of ultrasound in emergency medicine is provided in Table 1. The most cited publication published in Emergency Medicine Clinics of North America indicates the importance of using ultrasound to make a rapid and reliable diagnosis of the etiology of shock (13). Publications ranked two and three described the diagnostic value of echocardiography in prehospital care and the non-invasive bedside ultrasonographic measurement of low central venous pressure in emergencies (14, 15), which is consistent with the keyword “point-of-care ultrasound.”

In terms of journals, the impact factors were generally low (Figure 5A). Although there were few outstanding journals in this field, there was no drop in scholarly interest, as reflected by the rapid increase in the RRI (Figure 3A) in recent years. We consider there are two main reasons why it has received so much attention. On the one hand, as a convenient diagnostic tool, ultrasound is easier for people to accept than X-ray and CT because of its safety and radiation-free characteristics (25), especially for new-borns and pregnant women. These groups are more sensitive to radiation damage (26, 27). On the other hand, in the field of emergency medicine, the incidence of severe conditions, including abdominal trauma, deep vein thrombosis and pulmonary embolism, has been very high in recent years (23). In clinical practice, the rapidity of ultrasound examinations makes them very valuable.

### Research Focused on Ultrasound in Emergency Medicine

As shown in Figure 6B, pulmonary embolism is the keyword with the longest lasting popularity, as ultrasound is valuable for the diagnosis and treatment of this condition. A study has shown that venous thromboembolism manifesting as deep venous thrombosis or pulmonary embolism has a mortality rate of 6–12% (28). In a prospective multicentre trial, Mathis demonstrated a specificity of 95% for the diagnosis of peripheral pulmonary embolism by lung ultrasound (29). In terms of treatment, studies have shown that low-dose fibrinolysis under ultrasonic detection can reduce the risk of pulmonary hypertension in patients with pulmonary embolism (30). Therefore, it is understandable that using ultrasound to diagnose and treat pulmonary embolism has received much attention. However, in recent years, point-of-care ultrasound has been the focus of our research (Figure 6B).

We obtained maps of all keywords (Figure 6A) and analyzed the hotspots, and point-of-care ultrasound was the latest keyword. Because of its portability, point-of-care ultrasound plays an important role in emergency care, especially in the diagnosis of pleural effusions, lung consolidation and pneumothorax. These diagnostic accuracies range from 90 to 100% (31). A study from Korea has shown that for the diagnosis of acute ureter stones, point-of-care ultrasound is not only able to ensure the correct rate of diagnosis but also has the characteristics of being fast (32). This advantage can also be exploited at the diagnosis of acute heart failure (33), intussusception (34), thoracoabdominal injuries (35) and many other acute diseases. Because it is both fast and accurate, point-of-care ultrasound will become a future hotspot.

In addition, in terms of research fields, research is not limited to clinical medicine. Subjects related to clinical medicine, such as nursing, sports, and rehabilitation (Figure 7), are worthy of attention. We believe that the development of any field requires multidisciplinary communication and assistance, as does the use of ultrasound in the emergency medicine field.

## Limitations

This study investigated publications from the WOS database to obtain objective and reliable results. However, due to the limitation of the search to studies in English and the constant updating of the database, our results may differ slightly from the actual results. For more comprehensive results, databases such as Medline, Scopus or Google Scholar could be adopted and compared in further studies.

## Conclusion

The United States has contributed mostly to the field of use of ultrasound in emergencies medicine, and cooperation between countries is crucial. Furthermore, we predict that the total number of global publications will grow in the future. Importantly, our analysis reflected that pulmonary embolism was once the main research direction, but point-of-care ultrasound could be a research hotspot in the future. The collaboration of clinical medicine-related disciplines has played an important role in the development of this field. Overall, we believe our study provides profound insights into the current status of and the future trends of the use of ultrasound in emergency medicine.

## Data Availability Statement

The original contributions presented in the study are included in the article/Supplementary Material, further inquiries can be directed to the corresponding author.

## Author Contributions

SW, DX, and ZZ collected and analyzed the data and wrote the manuscript. JZ, WM, and YZ designed the study and revised the manuscript. SX provided the methodology. All authors read and approved the final manuscript.

## Funding

We acknowledge support from the National Natural Science Foundation of China (No. 81741111), the Changhai Hospital Medical New Technology Cultivation Project (No. NT201502), the Military Research and Logistics Equipment Project, and the Shanghai Health Planning Commission Funds, 2019 Shanghai Military-Civilian Integration Development Project.

## Conflict of Interest

The authors declare that the research was conducted in the absence of any commercial or financial relationships that could be construed as a potential conflict of interest.

## Publisher's Note

All claims expressed in this article are solely those of the authors and do not necessarily represent those of their affiliated organizations, or those of the publisher, the editors and the reviewers. Any product that may be evaluated in this article, or claim that may be made by its manufacturer, is not guaranteed or endorsed by the publisher.

## Abbreviations

WOS: Web of Science
RRI: relative research interest
GDP: gross domestic product
JCRs: journal citation reports
POCU: point-of-care ultrasound.
