# Supplementary material for: Mapping Trends and Hotspots Regarding the Use of Ultrasound in Emergency Medicine: A Bibliometric Analysis of Global Research
Source: Front Public Health. 2021 Dec 24;9:764642. doi: 10.3389/fpubh.2021.764642 (PMC8739757; doi:10.3389/fpubh.2021.764642)
Supplement: Supplementary file 1 [file Table_1.DOCX]

**Supplemental Table 1. The analytic consequence of 62 keywords with at least 56 occurrence times**

| **Label** | **Cluster** | **Occurrences** | | **Average appearing years  (AAY)** |
| --- | --- | --- | --- | --- |
| abdominal pain | 2 | 122 | 2014.8197 | |
| accuracy | 3 | 204 | 2015.25 | |
| adult patient | 1 | 71 | 2014.6761 | |
| age | 1 | 173 | 2015.0405 | |
| appendicitis | 3 | 92 | 2015.7826 | |
| article | 2 | 105 | 2015.2286 | |
| aware | 2 | 92 | 2017.3478 | |
| case | 2 | 514 | 2014.9436 | |
| case report | 2 | 195 | 2015.2154 | |
| cause | 2 | 130 | 2015.0769 | |
| comparison | 1 | 90 | 2015 | |
| complication | 2 | 156 | 2014.8397 | |
| condition | 2 | 142 | 2014.9225 | |
| confidence interval | 1 | 226 | 2014.5796 | |
| confidence interval ci | 1 | 62 | 2014.371 | |
| convenience sample | 1 | 95 | 2014.0842 | |
| ct scan | 3 | 262 | 2015.2951 | |
| data | 1 | 228 | 2015.0658 | |
| day | 2 | 120 | 2015.2833 | |
| diagnosis | 2 | 664 | 2015.0301 | |
| diagnostic accuracy | 3 | 112 | 2015.4821 | |
| difference | 1 | 124 | 2015.0403 | |
| effect | 1 | 106 | 2015.6509 | |
| emergency physician | 2 | 425 | 2015.0424 | |
| fast | 3 | 60 | 2013.7 | |
| feasibility | 1 | 58 | 2015.3793 | |
| group | 1 | 215 | 2014.8837 | |
| history | 2 | 117 | 2014.5897 | |
| january | 3 | 66 | 2014.8636 | |
| level | 1 | 151 | 2014.9272 | |
| literature | 2 | 134 | 2014.9552 | |
| management | 2 | 263 | 2015.3536 | |
| mean age | 1 | 76 | 2015.3421 | |
| measurement | 1 | 141 | 2014.8652 | |
| minute | 1 | 58 | 2014.4483 | |
| negative likelihood ratio | 3 | 60 | 2015.4667 | |
| negative predictive value | 3 | 103 | 2014.233 | |
| number | 1 | 123 | 2014.3577 | |
| observational study | 1 | 125 | 2014.936 | |
| pain | 2 | 192 | 2014.8646 | |
| participant | 1 | 65 | 2015.2769 | |
| physical examination | 2 | 91 | 2014.6484 | |
| pocus | 2 | 605 | 2017.777 | |
| positive predictive value | 3 | 80 | 2013.875 | |
| procedure | 1 | 171 | 2014.7544 | |
| prospective study | 3 | 88 | 2014.7841 | |
| radiologist | 3 | 81 | 2015.4938 | |
| range | 1 | 122 | 2015.0164 | |
| sensitivity | 3 | 365 | 2015.0575 | |
| significant difference | 1 | 78 | 2016.2051 | |
| sonography | 3 | 175 | 2013.7371 | |
| specificity | 3 | 338 | 2015.0118 | |
| study objective | 1 | 78 | 2014.3718 | |
| subject | 1 | 98 | 2014.6327 | |
| symptom | 2 | 112 | 2014.4464 | |
| systematic review | 3 | 56 | 2016.7321 | |
| total | 1 | 190 | 2014.9895 | |
| training | 1 | 172 | 2014.9302 | |
| trauma | 3 | 235 | 2014.0809 | |
| treatment | 2 | 184 | 2015.0707 | |
| year | 1 | 316 | 2014.8608 | |
| year old man | 2 | 61 | 2014.3443 | |
